# Vitrectomy With Silicone Oil Tamponade and Without Internal Limiting Membrane Peeling for the Treatment of Myopic Foveoschisis With High Risk of Macular Hole Development

**DOI:** 10.3389/fmed.2021.648540

**Published:** 2021-05-28

**Authors:** Yuou Yao, Jinfeng Qu, Xuan Shi, Jie Hu, Jing Hou, Heng Miao, Yong Cheng, Mingwei Zhao

**Affiliations:** ^1^Beijing Key Laboratory of Diagnosis and Therapy of Retinal and Choroid Diseases, Department of Ophthalmology, Peking University People's Hospital, Beijing, China; ^2^Eye Diseases and Optometry Institute, College of Optometry, Peking University Health Science Center, Beijing, China

**Keywords:** myopic foveoschisis, vitrectomy, silicone oil tamponade, internal limiting membrane, anatomical outcome

## Abstract

**Purpose:** To explore the efficiency and safety of the surgical procedure of pars plana vitrectomy (PPV) with silicone oil (SO) tamponade and without internal limiting membrane (ILM) peeling for myopic foveoschisis (MF) eyes with high risk of macular hole formation.

**Methods:** Three eyes (three patients) with MF and foveal detachment were enrolled into the study. Comprehensive preoperative ophthalmological assessments, including best corrected visual acuity (BCVA) and spectral-domain optical coherence tomography (SD-OCT) were performed on the eyes. Central foveal thickness (CFT) and thickness of continuous neurosensory retina at foveola were measured. All patients underwent PPV followed by SO tamponade and without ILM peeling. SO was removed when MF and retinal detachment were resolved. Patients were followed up postoperative at month 1, 3, 6, and 12.

**Results:** All the three eyes achieved complete resolution of MF and foveal reattachment with an average SO tamponade period of 11.67 ± 0.58 months. The average CFT at 6 months was 91 ± 27.5 μm, hence reduced significantly from baseline at 365.3 ± 137.85 μm (*P* = 0.037). There was no postoperative macular hole formation despite the average preoperative sensory retina thickness of 58 ± 20.07 μm. Mean BCVA was improved from logMAR 1.43 ± 0.75 to logMAR 0.8 ± 0.75 on the last follow-up. Manageable SO-related complications were reported, including SO emulsification, ocular hypertension, and cataract.

**Conclusion:** Vitrectomy with SO tamponade and without ILM peeling as an optional surgical protocol to treat MF is effective and safe, especially for MF eyes vulnerable to macular hole formation.

## Introduction

Myopic foveoschsis (MF) is one of the major causes of impaired vision in highly myopic eyes, which affects 9–34% patients with high myopia (1, 2). Although MF in general progresses slowly and most patients retain relatively good vision, half to two-thirds of the MF patients will develop MH or retinal detachment within 2 years (3, 4). Pars plana vitrectomy (PPV) combined with ILM peeling followed by gas tamponade is the most common surgical treatment for MF. Postoperative MH is a common complication that occurs in 5–28% of surgical cases with poor visual outcome (5–9). However, for MF eyes combined with foveal detachment (FD), which often reveal an extremely thin continuous sensory retina on an optical coherence tomography (OCT) image, ILM peeling could result in the formation of MH. Thus, we introduce PPV without ILM peeling and prolonged SO tamponade period for MF eyes with thin continuous sensory retina or FD that are vulnerable to postoperative MH formation (10).

## Methods

All methods in this study were conducted according to the Declaration of Helsinki. The ethics approval of this study was obtained from the Institutional Review Board of the Peking University People's Hospital. This study was a small prospective interventional case series, which enrolled three eyes of three consecutive MF patients with FD at the Peking University People's Hospital. All the three patients experienced progressively reduced vision or metamorphopsia, which were attributed to MF. The eyes with preoperative full-thickness MH, myopic choroidal neovascularization that could affect the central vision, and eyes with a history of other ocular fundus diseases were excluded. Before the surgical procedure, informed consent was obtained from the study subjects.

### Preoperative Assessment

The following data were collected: age, sex, preoperative lens status, refractive error, axial length, and preoperative best corrected visual acuity (BCVA) (Table 1). Thorough fundus examination using indirect binocular ophthalmoscopy and foveal microstructures scanned using a spectral-domain OCT (SD-OCT, Optovue, Fremont, CA, US or Carl Zeiss Meditec) were performed to confirm the presence of MF. SD-OCT was performed over a retinal area of 6.0 × 6.0 mm using a radial scan. The FD and central foveal thickness (CFT) were visualized on all the lines of the radial scan images. CFT was measured at the highest foveal point of MF. Thickness of the continuous neurosensory retina at foveola was also determined based on the OCT image.

**Table 1:** Demographic, ophthalmological, surgical treatment, and follow-up data of the patients.

**Patient no**.	**Age (years)**	**Lens status**	**Refraction error (diopters)**	**Axial length (mm)**	**Surgical treatment**	**SO endotamponade period**	**SO related complications**	**Pre-operative BCVA (logMAR)**	**Post-operative BCVA (logMAR)**	**Follow-up period after SO removal**	**Total follow-up period**
1	47	Pseudophakic	−10	28.01	PPV + SO tamponade	11 months	SO emulsification	1	0.7	12 months	23 months
2	60	Pakic	−14	28.15	PPV + SO tamponade	12 months	Ocular hypertention; complicated cataract	2.3	1	3 months	15 months
3	65	Phakic with lens opacity	−12	30.85	PPV + SO tamponade with phacoemulsification and IOL implanation	12 months	None	1	0.7	3 months	15 months

### Surgical Procedure

A standard 25-gauge, three-port PPV was performed by a single experienced surgeon (MZ). Patients with apparent lens opacity underwent phacoemulsification with implantation of intraocular lens surgery at the same time. After completion of the core vitrectomy, triamcinolone acetonide (0.1–0.2 mL; 40 mg/mL) was injected to identify and help achieve complete detachment of the posterior hyaloid from the posterior surface of the retina. The ILM was not peeled. Fluid–gas exchange was performed, followed by SO injection. Patients were instructed to maintain face-down position for 3 weeks following surgery and afterward maintain a face-down position 2 h a day until foveal reattachment.

### Postoperative Assessment

Full ophthalmological examinations, including BCVA and SD-OCT, were performed at months 1, 3, 6, and 12. SO was removed until both the foveoschisis cavity and FD disappeared on all the radial SD-OCT scans. Full ophthalmological examinations and SD-OCT were performed at least 3 months following SO removal.

### Anatomical and Functional Outcome Measures

Anatomical outcomes were assessed by SD-OCT morphological changes, including foveal reattachment, CFT, and resolution of foveoschisis cavity. Functional outcome was assessed by BCVA in logarithm of the minimum angle of resolution (logMAR).

### Statistical Methods

Statistical analyses were carried out using the SPSS software package, V.22.0. Quantitative data were presented as mean ± SD for parametric data.

## Results

**Figure 1:**
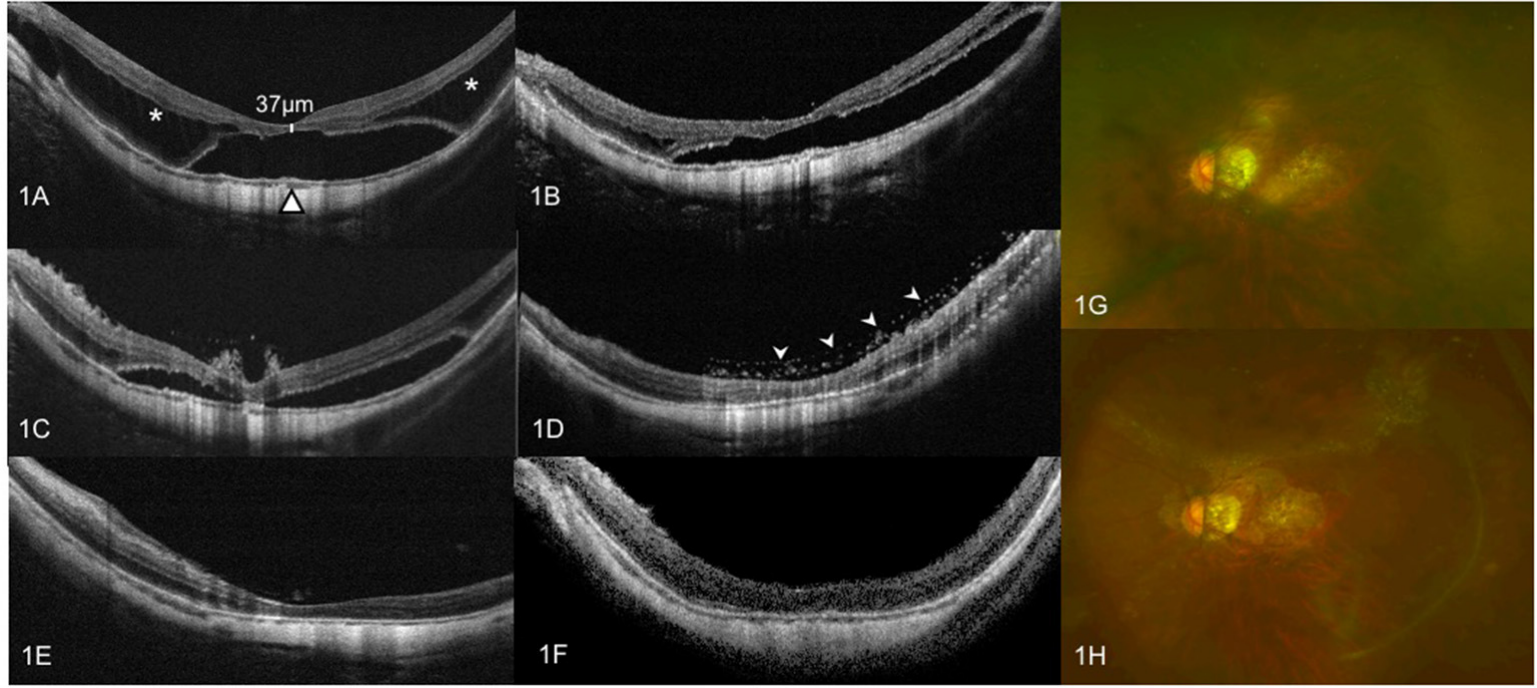
OCT images of case 1. Left eye with a refractive error (spherical equivalent) of −10.0 diopters in a 47-year-old female: **(A)** Preoperative OCT image showing outer retinoschisis (asterisks) and FD (triangle), combined with a thickness of 37 μm of continuous neurosensory retina. **(B)** OCT image at 3 months after vitrectomy with SO tamponade showing a moderate foveoschisis and FD resolution. **(C)** OCT image at 6 months after vitrectomy with SO tamponade showing a remarkable foveoschisis and FD resolution. **(D)** OCT image at 11 months after vitrectomy with SO tamponade shows complete foveoschisis and FD resolution, with emulsified SO droplets on the surface of macular (arrow heads). **(E)** OCT image at 1 week after SO surgical removal does not show MF relapse. **(F)** OCT image at 12 months after SO surgical removal does not show MF relapse. **(G)** Preoperative color fundus image. **(H)** Color fundus image at 11 months after vitrectomy with SO tamponade showing emulsified SO droplets on the surface of retina.

**Figure 2:**
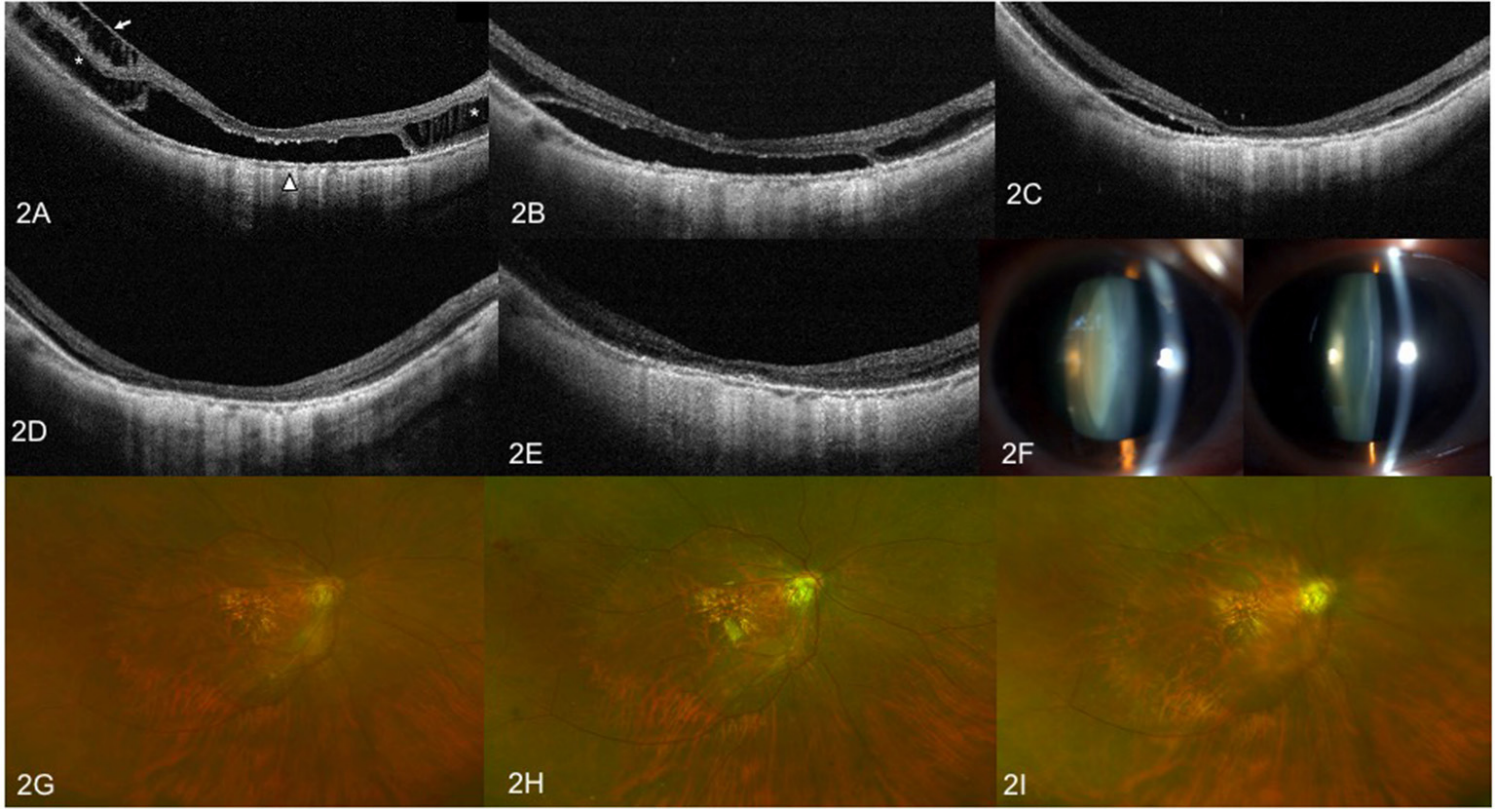
OCT images and anterior segment photographs of case 2. Right eye with a refractive error (spherical equivalent) of −14.0 diopters in a 60-year-old female. **(A)** Preoperative OCT image showing outer retinoschisis (asterisks), inner retinoschisis (arrow), and foveal detachment (triangle). **(B)** OCT image at 3 months after vitrectomy with SO tamponade showing slight resolution of the inner retinoschisis, but no change in outer retinoschisis and FD. **(C)** OCT image at 6 months after SO tamponade showing remarkable foveoschisis and FD resolution. **(D)** OCT image at 12 months after vitrectomy with SO tamponade showing complete foveoschisis and FD resolution. **(E)** OCT image at 3 months after SO surgical resection does not show MF relapse. **(F)** Anterior segment photograph of bilateral eyes at 12 months after SO tamponade. The left image shows visually significant nuclear sclerotic cataracts of the right eye, when compared to contralateral eye. **(G)** Preoperative fundus image. **(H)** Color fundus image at 1 month after vitrectomy with SO tamponade. **(I)** Color fundus image at 3 months after SO removal surgery.

**Figure 3:**
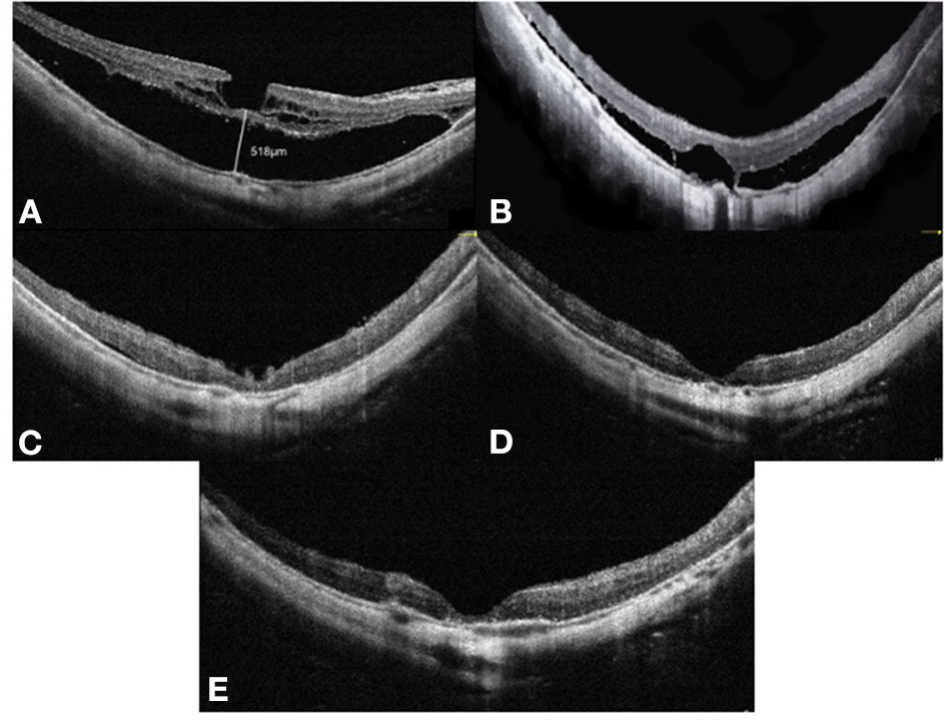
OCT images of case 3. Right eye with a refractive error (spherical equivalent) of −12.0 diopters in a 65-year-old female: **(A)** Preoperative OCT image showing retinoschisis and foveal detachment, with a height of 518 μm. **(B)** OCT image at 3 months after vitrectomy with SO tamponade showing a moderate foveoschisis and FD resolution. **(C)** OCT image at 8 months after SO tamponade showing a remarkable foveoschisis and FD resolution. **(D)** OCT image at 12 months after vitrectomy with SO tamponade showing a complete foveoschisis and FD resolution. **(E)** OCT image at 3 months after SO surgical removal does not show MF relapse. OCT, optical coherence tomographic; FD, foveal detachment; SO, silicone oil.

This study included three eyes (three patients) with symptomatic MF. Demographic, ophthalmological, surgical treatment, and the follow-up data of the patients are presented in Table 1. All the patients were female, with a mean age of 57.3 ± 9.29 (47–65) years. The mean preoperative BCVA was logMAR 1.43 ± 0.75 and improved to logMAR 0.8 ± 0.75 on the last follow-up visit. The average thickness of sensory retina was 58 ± 20.07 μm (37–77 μm), and the average CFT was 365.3±137.85 μm (250-518 μm). Patients 1 and 2 achieved foveola retinal reattachment at 6 months after the surgery (Figures 1, 2). Patient 3 did not show up at 6 months follow-up; however, the 3 months follow-up visit postoperatively showed foveola retinal reattachment, and the 8-month visit showed foveal retinal reattachment except with the slight detachment on the temporal side of the foveola on the OCT image (Figure 3). In addition, the average CFT was 91 ± 27.5 μm (67–121 μm) on the 6 months visit of Patients 1 and 2 and 8-month visit of Patient 3. The CFT of 6–8 months reduced significantly compared with baseline (*P* = 0.037). The SO tamponade period was 11 months for Patient 1, and 12 months for Patients 2 and 3, with an average SO tamponade period of 11.67 ± 0.58 months, the MF and FD resolved completely. The mean follow-up period was 17.67 ± 4.62 months (15–24 months), during which two patients experienced complications during the SO tamponade period. At 6 months follow-up, SO emulsification was observed in Patient 1, which at the 11-month visit showed progression, though without ocular hypertension. Patient 2 experienced ocular hypertension at 2 weeks after SO tamponade until 4 months, which was controlled by topical anti-glaucoma medications. She also developed visually significant cataract at 12 months after SO tamponade, and received SO removal surgery combined with cataract surgery. All patients retained stable macular microstructure without retinoschisis and FD relapse at least 3 months after SO removal surgery, and Patient 1 was followed 12 months after SO removal.

## Discussion

There is no consensus regarding surgical indications for MF; however, many patients experience slow, progressive visual deterioration, and may exhibit a stable visual acuity for years. Moreover, spontaneous resolution of MF is rare, with various studies reporting on specific cases (11–15). From among 207 MF eyes without FD or lamellar MH, Shimada et al. (3) reported a spontaneous resolution rate of 3.7%. They also considered that eyes with extensive macular retinoschisis and posterior hyaloid are more likely to progress, rather than remain stable or exhibit a spontaneous resolution. Studies have concluded that symptomatic MF, especially FD cases, require surgical interventions to prevent the development of full-thickness macular holes (FTMH) or macular hole retinal detachment (MHRD) (5, 6). In this study, we included MF eyes with FD and progressive visual acuity deterioration.

The exact mechanisms of MF have not been elucidated; however, it is more likely to develop from complex tractional forces from adherent vitreous cortex, rigid ILM, potential retinal arterioles, pathological axial length elongation, and posterior staphyloma (16, 17). Therefore, the tractional force generated by vitreous and ILM can be relieved by PPV combined with ILM peeling to treat MF (7). When used to treat symptomatic MF patients, PPV with or without ILM peeling followed by gas tamponade has been reported to yield good anatomical and functional results. This is the most widely performed surgery (18–21).

One serious complication that is associated with vitrectomy for MF is postoperative FTMH and MHRD. Moreover, as mentioned above, MF eyes have more rigid ILM than non-myopic eyes, which is vital for MF pathogenesis. The removal of rigid ILMs in MF eyes is technically challenging, and has been correlated with postoperative FTMH (9). Epidemiologically, FTMH has been reported to occur in 5–28% of the eyes subjected to PPV with ILM peeling for MF, leading to poor visual prognosis (5–9). Since Shimada et al. (8) proposed the fovea-sparing ILM peeling technique to inhibit postoperative FTMH development, studies comparing this technique and the traditional non-fovea-sparing ILM peeling technique have reported inconsistent conclusions. Most studies reported that fovea-sparing ILM peeling inhibited intraoperative or postoperative FTMH formation, but enhanced the occurrence of postoperative contractions of the remaining ILM (0–60%) (8, 20, 21). A few studies concluded that traditional ILM peeling can achieve an FTMH formation rate that is comparable to that of fovea-sparing ILM peeling (22). Gao et al. (10) showed that defects of the inner segment/outer segment junction, including FD, increased the risk of postoperative FTMH and MHRD. MF eyes with extremely thin continuous sensory retina, which are caused by elongation of the sclera, such as the eyes in the present study, are predisposed to postoperative MH development, especially after ILM peeling. Previous studies had various inclusion criteria of MF patient enrollment, with few including accurate thickness of the thinnest sensory retina. Wang et al. (23) have reported that foveal distortion, especially disoriented foveal Müller cell fibrils, can cause visual impairment in MF patients, indicating that the key to restoring the BCVA of MF patients might be flattening retinoschisis. It has also been suggested that ILM preservation may prevent foveola degeneration, for ILM is part of the Müller cell fibrils (24). Thus, it is reasonable to postulate that for patients who are predisposed to iatrogenic FTMH, ILM peeling should be avoided.

Vitrectomy with gas or balanced saline solution tamponade for MF without FTMH have been performed with variable high success rates (75–100%). Gas tamponade exhibited good outcomes on BCVA improvement or anatomical resolution (18, 25, 26), possibly by inducing retinal repositioning by pushing back the retina while keeping the retina surface dry. However, the SO used in vitrectomy without ILM peeling for MF without FTMH has not been scientifically evaluated. Hattori et al. (9) documented that surgeons prefer extensive and long-lasting tamponade materials for MF eyes with more severe myopic tractional maculopathy, such as MF with FD or lamellar MH.

Therefore, studies should aim at developing approaches to safely relieve the tangential traction of the rigid ILM, while tightening the force to neurosensory retina caused by elongation of the sclera without developing postoperative FTMH or MHRD. Even though SO has a smaller surface tension than gas, we found that as long as it is in vitreous cavity, it has the ability to provide a sustainable force for up to an average of 11.67 months of tamponade period. The time taken to achieve foveola reattachment after SO tamponade for three patients was around 6 months, while full retinal reattachment was achieved at around 12 months. In this study, the longer SO tamponade period led to several SO-related complications, such as emulsification, ocular hypertension, and cataract development. However, these complications can be chemotherapeutically or surgically regulated and do not necessarily leave permanent damage. Even though we enrolled high-risk FTMH formation (average 58 μm of sensory retinal thickness) MF patients, no patient in our study developed postoperative MH, thereby proving that our surgical strategy is relatively safe. All eyes achieved retinal reattachment, retinoschisis resolution, and improvement of BCVA, suggesting that the surgical protocol was effective.

The major limitation of this study is the small sample size. Furthermore, the absence of objective examination of macular functions such as multifocal electroretinogram and/or microperimetry was another limitation. Nevertheless, to the best of our knowledge, this is the first report on PPV with SO tamponade but without ILM peeling surgery to treat myopic foveal schisis eyes with a high risk for MH development. Based on our preliminary findings, this surgical technique might be easy to perform, effective for retinoschisis, and safe for preventing FTMH and MHRD, which is an optional surgical protocol for the treatment of MF. However, larger cohort studies with long-term follow-up periods are needed to confirm the effectiveness and safety of this surgical protocol.

## Data Availability Statement

The raw data supporting the conclusions of this article will be made available by the authors, without undue reservation.

## Ethics Statement

The studies involving human participants were reviewed and approved by Institutional Review Board of the Peking University People's Hospital. The patients/participants provided their written informed consent to participate in this study.

## Author Contributions

YY and MZ designed this study and wrote this article. YY, JQ, XS, YC, JHo, and HM collected and measured data. JHu and YY analyzed data. All authors discussed the results and commented on the manuscript.

## Conflict of Interest

The authors declare that the research was conducted in the absence of any commercial or financial relationships that could be construed as a potential conflict of interest.
